# Defining a Core Genome for the Herpesvirales and Exploring their Evolutionary Relationship with the Caudovirales

**DOI:** 10.1038/s41598-019-47742-z

**Published:** 2019-08-05

**Authors:** Juan S. Andrade-Martínez, J. Leonardo Moreno-Gallego, Alejandro Reyes

**Affiliations:** 1 Research Group on Computational Biology and Microbial Ecology, Department of Biological Sciences, Universidad de los Andes, Bogota, Colombia; 2 Max Planck Tandem Group in Computational Biology, Universidad de los Andes, Bogota, Colombia; 3 Centre for Genome Sciences and Systems Biology, Department of Pathology and Immunology, Washington University in Saint Louis, Saint Louis, MO 63108 USA

**Keywords:** Herpes virus, Viral evolution, Phylogeny, Classification and taxonomy, Phylogenetics

## Abstract

The order Herpesvirales encompasses a wide variety of important and broadly distributed human pathogens. During the last decades, similarities in the viral cycle and the structure of some of their proteins with those of the order Caudovirales, the tailed bacterial viruses, have brought speculation regarding the existence of an evolutionary relationship between these clades. To evaluate such hypothesis, we used over 600 Herpesvirales and 2000 Caudovirales complete genomes to search for the presence or absence of clusters of orthologous protein domains and constructed a dendrogram based on their compositional similarities. The results obtained strongly suggest an evolutionary relationship between the two orders. Furthermore, they allowed to propose a core genome for the Herpesvirales, composed of 4 proteins, including the ATPase subunit of the DNA-packaging terminase, the only protein with previously verified conservation. Accordingly, a phylogenetic tree constructed with sequences derived from the clusters associated to these proteins grouped the Herpesvirales strains accordingly to the established families and subfamilies. Overall, this work provides results supporting the hypothesis that the two orders are evolutionarily related and contributes to the understanding of the history of the Herpesvirales.

## Introduction

It is estimated that, for any given community, the number of viral particles is 10 to 100 times higher than the number of prokaryotic organisms^1^. Apart from their abundance, viruses are also characterized by their high diversity in terms of structure, genome size, strategies for replication and expression, and morphology of the virion^1,2^. Moreover, they display high infection and mutation rates^3^. Such high mutation rates and diversity are a hurdle to efforts aimed at reconstructing the evolutionary history of these elements. The situation is entangled even further in bacterial viruses (phages), where distant relationships are clouded by horizontal gene transfer (HGT)^4^. Nevertheless, advances have been made in elucidating the connection between major groups of viruses, especially establishing the relationships of lineages with eukaryotic and prokaryotic hosts. In the realm of dsDNA viruses, two notable examples are the connection between the Polintoviruses and phages of the family Tectiviridae; and the common ancestor between Herpesvirales and Caudovirales^1^.

Viral classification approved by the ICTV has been based on morphology, life-style, host range, and type of nucleic acid^5^. The order Herpesvirales is composed by dsDNA viruses, most of them with vertebrate hosts^6^, and comprises important human pathogens such as the Epstein-Barr virus, the Human Cytomegalovirus and the Varicella-Zoster virus. Analyses of the human blood virome have found a high prevalence of members of such clade in healthy human individuals^7^. In fact, it is predicted that some species may establish chronic infections in up to 90% of the human population^8^, affecting both the immune system and gene expression in certain organs^9^.

Structurally, the Herpesvirales are characterized by a common morphology: an icosahedral capsid, which encloses the DNA, surrounded by a layer of tegument and an envelope^10^. At the genetic level, for the most abundant family, the Herpesviridae, a core genome (i.e.: a set of genes harboured by the common ancestor of the clade) composed by 43 genes with high conservation among the subfamilies, grouped either by function or location in the mature virion, has been established^11^. In spite of this, considering the order as a whole, the only proven case of conservation is that of the ATPase subunit of the DNA terminase, a protein involved in the packaging of DNA during virion maturation^12^.

Interestingly, such subunit is also present in the Caudovirales, the dsDNA tailed phages, with evidence of conservation with the Herpesvirales both in the amino acidic sequence and protein structure^6,13,14^. However, multiple sequence alignments of these peptide show that identity and similarity are scarce (see Fig. 6 in^12^). This is true when comparing between the orders as well as within the Herpesvirales. Another example of structural similarity with the Caudovirales is the major capsid protein, which contains, in both clades, the HK-97 fold^15^. Furthermore, there are notable parallelisms in the viral cycles and the processes for genome packaging and delivery into the host for both orders^16^.

To tackle the hurdle that high mutation rates of viruses impose into the analyses of their evolutionary relationships, two approaches have been proposed in recent years. The first one involves obtaining the distribution of k-mers of a given predefined size in the whole genome, and subsequently constructing groups based on the similarity of this distribution^17^. The second one identifies clusters of either orthologous genes or domains, in fully sequenced phage genomes. These are then employed to group prokaryotic viral genes or domains into phage orthologous groups, or POGs^4,18^. The importance of considering domains, and not full multidomain proteins, stems from the fact that the evolutionary histories of different domains might vary, even if they belong to the same peptide^18^. Note that if this procedure is applied to a group of viruses with a common origin, it is possible to determine which orthologous domains are representative of that taxon, making it possible to determine whether other, unclassified samples, belong to that clade or not.

In this work, the second method was modified to be applied to viruses with eukaryotic hosts, and to consider protein (amino acid) sequences instead of DNA, so as to define clusters of Viral Domain Orthologous Groups (VDOGs), which could be used as taxonomic signatures of viral clades. We used these conserved protein domains as markers to elucidate the evolutionary relationship of the Herpesvirales and Caudovirales. By taking such approach, we were not only able to provide evidence for an evolutionary relationship among these lineages, but also postulate two hypotheses regarding that relationship. We further propose, for the first time, a core genome for the Herpesvirales order, composed of 4 proteins: ATPase subunit of the DNA terminase, dUTP-diphosphatase, ribonucleotide reductase, and the catalytic subunit of the DNA polymerase, all related either to viral DNA synthesis or DNA packaging in the mature virion.

## Methods

### VDOG construction

Groups of orthologous domains were built following the methodology proposed by Kristensen *et al*.^18^ considering both bacteriophages (viruses with bacterial and archaeal hosts) and viruses with eukaryotic hosts. Briefly, complete viral genomes were downloaded (April 2015) from the National Institute of Health (NCBI) public databases *GenBank* and *RefSeq* using the query (((((*viruses*[*Organism*]) *NOT cellular organisms*[*Organism*]) *AND srcdb_refseq*[*Properties*]) *NOT vhost bacteria*[*Filter*]) *AND* “*complete genome*”) for viruses with eukaryotic hosts and (((((*viruses*[*Organism*]) *NOT cellular -organisms*[*Organism*]) *AND srcdb_refseq*[*Properties*]) *AND vhost bacteria*[*Filter*]) *AND “complete genome”*) for phages. So as to remove sequences not corresponding to complete genomes, additionally all entries with the terms “segment”, “ORF”, “Gene”, “mutant”, “protein”, “complete sequence”, “Region”, “CDS”, “UTR”, “recombinant” or “terminal repeat” were removed.

This search yielded a total of 42,741 sequences. For genome dereplication, a two-step process was designed. First, genomes which shared identity of at least 95% over their full length were clustered using CD-HIT^19,20^. Next, the representative sequences of these genome clusters (i.e.: the longest in each group) were dereplicated at the proteome level. To accomplish this, their correspondent proteomes were predicted using Prodigal^21^ and GenMark^22^, within the tool RASTtk^23^. The proteomes of all representative genomes were compared all against all using CDHIT, and proteins with more than 95% similarity over their total length were considered homologous and thus clustered in the same group. With the generated protein clusters, a matrix of presence/absence of each cluster in each proteome was constructed. This matrix was used for the proteome dereplication itself. In this step, two proteomes were clustered together if one of three conditions was fulfilled: (i) both had less than 20 proteins and shared all of them, (ii) both had more than 20 proteins and they shared 90% of their proteins, or (iii) only one had less than 20 proteins and it shared all its proteins with the other. A representative proteome (the one with the most proteins and, if possible, an associated RefSeq genome) was selected from each proteome cluster and used for further analyses.

After dereplication, 13,922 proteomes were deemed representative. Protein domains from this sequences were identified with InterPro Scan^24^, with multidomain proteins being split into their constituent domains. Protein regions larger than 40 amino acids that did not contain any known domain were kept as well. Together, the protein domains and other non-annotated protein regions were used to build orthologous groups using CogSoft^25^ with an e-value of 10^−5^ and minimum coverage of 50% for BLAST analyses prior to orthologous group construction. The generated clusters of viral domain orthologous groups (VDOGs) were used to create Hidden Markov Models (HMM) of each VDOG with HMMER^26^, and the resulting models were concatenated into an HMM database by employing the same tool.

For each VDOG, information was recorded on the sequences that compose it and the accession number of the corresponding genomes in NCBI. The location of each domain or region within its full peptide is also recorded.

### Complementing herpesvirales proteomes

The initial search and dereplication for the construction of the VDOGs yielded just over 380 representative proteomes from the order Herpesvirales. In order to increase and update the quantity of sequences considered for further analyses, a new search was conducted on December 2016 of the *genome* and *nucleotide* public databases of the NCBI, using as keywords the taxonomy IDs of the order Herpesvirales and its families: 548681 for the Herpesvirales, 10292 for the Herpesviridae, 548682 for the Alloherpesviridae and 548685 for the Malacoherpesviridae. For the nucleotide database, the term “whole coding sequence” was also used in the search.

The genomes of this taxonomical order range in size from 125 to 290 kbp^27^. With this as a benchmark, the sequence length of the hits obtained was verified to filter entries that were too short (only a few kilobases long) to constitute either whole genomes or complete coding sequences. The two-step genome and proteome dereplication process was carried out in exactly the same way as described above, leading to a final number of 541 dereplicated Herpesvirales proteomes.

For the Caudovirales, the initial search and dereplication yielded 2045 representative proteomes. Note that the ICTV nomenclature is revised yearly, and the number of subfamilies and families of the order Caudovirales has expanded since 2015^28^. However, with the exception of 9 members of the subfamily Sepvirinae of Podoviridae, none of the representative proteomes analysed in this study (see below) were registered yet in NCBI as members of these new clades. Hence, the taxonomy employed here is adjusted to the revision of 2014, ratified in 2015^29^, which was the most recent one when the initial search was conducted.

### Detection of VDOGs in viral proteomes

Using the HMMs generated for each VDOG and the function hmmscan from HMMER^26^, every protein from every Caudovirales and Herpesvirales representative proteome was scanned (all 2045 and 541 respectively). If it produced a hit against the HMM of a given VDOG with an e-value lower than 1e-10, it was determined that the protein harboured the domain associated with that orthologous cluster, and, by extension, so did the proteome and its associated genome.

A special procedure was carried out for the Herpesvirales with VDOG 105, associated with the ATPase subunit of the terminase. Even though this is the only peptide with proven conservation among all the Herpesvirales^12^, its correspondent VDOG was only detected sparsely in the Alloherpesviridae, Malacoherpesviridae, Gammaherpesvirinae and the unclassified Herpesvirales when screening their representative proteomes against the HMM database.

To analyse this cluster of proteins, a BLAST database was created with the original sequences that belong to that VDOG. Each protein sequence of the representative proteomes belonging to the Herpesvirales was compared against the database; a given proteome was determined to harbour the domain associated to that VDOG if a hit was obtained for at least one protein that had at least 50% similarity in 50% of the length of the complete protein with an e-value lower than 1e-10, or had an alignment length of more than 100 amino acids with at least 60% similarity with an e-value lower than 1e-10.

The former thresholds were derived from the fact that the shorter sequences that originally belonged to VDOG 105 were around 100 amino acids long. Nevertheless, when considering hits with alignments that spanned 50% or more of the whole protein, the criterion was relaxed to 50% similarity since the location and length of the domains of interest in the new sequences (the ones that were not used to generate the HMM) were unknown at that point.

### Selection of prevalent VDOGs

Prevalent Herpesvirales VDOGs (i.e.: those present in the majority of the selected viral proteomes from the order), are those which are found in at least 60% of the dereplicated proteomes of the families Herpesviridae (n = 539), Alloherpesviridae (n = 19) and Malacoherpesviridae (n = 3), as well as in one or both of the two unclassified species within the order. These VDOGs were identified using the information, obtained above, of presence or absence of each orthologous protein cluster in each proteome.

To visualize their distribution in the Herpesvirales, a heatmap of presence/absence was generated with these prevalent VDOGs. An additional heatmap was constructed considering Caudovirales proteomes, so as to determine if any of those VDOGs was also prevalent in this order or any of its subclades. The protein associated to each one of the prevalent VDOGs was determined based on a manual revision of the annotation of the sequences that composed it.

### Selection of representative VDOGs

To determine which VDOGs were representative (i.e.: taxonomic firms of a given clade), three criteria were considered: (i) the sum of the negative log2 of the precision (TPR, or true positive rate) and the negative log2 of the sensitivity (1-FPR, or 1 – false positive rate) that each VDOG achieved when being employed in a binary classification for all viruses of interest in their clades, where values close to zero are indicative of both high precision and sensitivity. Here, a binary classification is the process of classifying a virus as member of a given clade or not (e.g.: Caudovirales or non-Caudovirales, Herpesviridae or non-Herpesviridae, etc.). A VDOG fulfilled this criterion for a given taxonomic classification if it had a precision-sensitivity value equal or lower than 2. (ii) Mutual information, an entropy-based measure that allowed selecting those VDOGs that were prevalent and unique for each clade. A VDOG fulfilled this criterion if it had a modified Z-score equal or higher than 3.5 on a normalized mutual information distribution for VDOGs in that clade, which implies an anomalous value under Iglewicz & Hoaglin^30^. (iii) Performance as features of a random forest classifier. For this last criterion, VDOGs were evaluated as features of a random forest classifier, trained using Scikit-Learn^31^, with 60 trees. The classifier determined the clade, either order or family, associated with a given proteome based on the presence or absence of the orthologous protein clusters in the latter. Those VDOGs that were used as features in the best classifier as measured by its accuracy on a test set (made up of 50% of the original data) were selected and said to fulfil the criteria.

Representative VDOGs were determined for the order and family taxonomic ranks using the representative proteomes from the Herpesvirales and Caudovirales. In that context, representative VDOGs are those which fulfil criterion (iii), and either or both (i) or (ii) at the desired taxonomic level.

Note that, based on the definitions given above for prevalent and representative VDOGs, these two classifications are independent, thus neither mutually exclusive nor interchangeable. However, a VDOG related to a gene associated with a core genome should be both prevalent and representative, implying it is present in the majority of the proteomes, and by extension genomes, and being a taxonomic marker for that clade, which is expected since it derives from the common ancestor of the clade.

### Dendrogram of VDOGs for the two orders

Based on the representative VDOGs obtained, a new matrix of presence/absence of each cluster in each proteome was constructed. For every pair of proteomes, the Jaccard distance was calculated, and a dendrogram constructed based on such parameter. Proteomes from the dataset originally used to generate the VDOGs were used as outgroups, specifically from the eukaryote-infecting order Nidovirales (n = 513), ssDNA family Parvoviridae (n = 172), and dsDNA family Adenoviridae (n = 242). The Nidovirales were chosen since they are the viral group with RNA as nucleic acid closest to the Caudovirales and Herpesvirales branch in the dendrogram constructed through k-mer frequencies by Zhang *et al*.^17^. The Parvoviridae, as well as other ssDNA viral families, seem to have arisen from bacterial plasmids and RNA + viruses, thus being completely unrelated to the dsDNA phages^1^. Finally, the Adenoviridae are one of the dsDNA family groups which compose the other major dsDNA viral lineage, comprising, among other clades, the Tectiviridae and Polintoviruses^2^.

### Phylogenetic tree of the Herpesvirales

The criteria outlined above for prevalent VDOGs was fulfilled by 5 orthologous protein groups, which were also deemed representative VDOGs, as they fulfilled the criteria outlined above, making their associated proteins candidate members of the core genome of the Herpesvirales. Thus, a phylogeny was constructed based on their composing sequences. Note that since VDOGs are associated to domains within proteins, the process was carried out mostly with segments of proteins, instead of full sequences, from representative Herpesvirales proteomes. This also implies that, for some cases, more than 1 region in a given protein could produce a hit with the HMM of a VDOG. To tackle this, only the hit with the lowest independent e-value was selected per protein, as measured by the function hmmscan of HMMER^26^.

For cases in which there were hits in multiple proteins, a BLAST database was constructed with the sequences already selected. Then, each of the potential segments was compared against the database, restricting the results to 1 hit per database sequence. The product of the similarity and percentage of coverage for each alignment was calculated and summed over all hits; the sequence with the highest score after the summation was employed.

The tree was constructed using 56 Herpesvirales proteomes selected randomly from the ones available. The specific number per family, and in the case of Herpesviridae per subfamily, was balanced based on the relative abundance of representative proteomes of that group compared to the total number of proteomes for the order. The number of sequences from proteomes of the same viral strain was restricted to one.

The selected sequences were aligned using Muscle^32^. Gapped regions at the beginning and end of the alignments were edited with Jalview^33^. Afterwards, an evolutionary model for each orthologous protein cluster was selected with ProtTest^34^. Following, the alignments were concatenated and a phylogenetic tree constructed by maximum-likelihood with RaxML^35^, with 100 steps of gradient descend and 1,000 bootstraps. In this case, three randomly selected Myoviridae proteomes that harboured all the analysed VDOGs were used as outgroups, since no other eukaryotic viral group contained all the studied orthologous clusters on their proteomes. Only proteomes which contained all selected VDOGs were considered.

### Synteny analysis of prevalent VDOGs

The preponderance of HGT in distantly related phages led us to consider the location of the proposed genes as a measure of their evolutionary conservation. Since HGT, which is frequent in the Caudovirales^36^, consists in the exchange of given fragments of DNA, it follows that genes clustered together in the genome are more likely to be transferred in block, and thus have similar evolutionary histories. Furthermore, in the Herpesviridae, gene dispositions of the members of the core genome are known to be conserved between subfamilies^6^.

To determine whether there is any clustering of the genes associated with the five prevalent VDOGs in the genomes, which would be suggestive of conservation in the order and direction (synteny) of those genes, the distance in genes spanning each possible combination of 2, 3, 4, or all 5 prevalent VDOGs in each genome was calculated. A conservative approach was taken when measuring this distance for three or more VDOGs: albeit when considering two VDOGs the minimum distance found between any two copies was measured, for three or more the distance was defined as the maximum number of genes between the furthest separated copies of different VDOGs, such that all other copies, if any, of the evaluated VDOGs were encompassed between the two selected. This measurement is then contingent both to genome size and the number of repeats of each gene. Furthermore, since sequencing of circular genomes can be deposited in arbitrary origins, all genomes were assumed circular and the calculation of the distances was corrected for either direction.

Analyses were carried at the family taxonomical level. For each case, a null distribution with 10,000 data points was established. To construct this null distribution, a dummy genome, represented as a list of integers between 0 and 5, was generated, where numbers 1 to 5 represented one of the genes associated to the prevalent VDOGs and 0 any other gene. The size of this genome was equal to the median genome size (in genes) for the family evaluated. Regarding the number of gene copies, the maximum number of copies found in any genome from the clade in question was employed, since it will reduce the expected distance among VDOGs, thus making the comparison more stringent. The position of the genes was randomly shuffled 10,000 times and the distances between genes of interest was measured in the same way described above.

The generated null distribution was compared to the one obtained for each pair of VDOGs in three ways: first, a two-sided Mann-Whitney U test was performed to compare the medians, followed by a two-sided Kolmogorov-Smirnov test to compare the shape of the distributions, both with a significance level of 1%. Finally, a more restrictive randomization test was performed where it was determined whether the median of the real distribution was smaller than the 1^st^ percentile of the null distribution. A set of genes was considered to be located closer than expected by chance if the first two analyses yielded a p-value of 0.01 or less, and if the median of the distance was smaller than the 1^st^ percentile of the null distribution. The election of the non-parametric Mann-Whitney U and Kolmogorov-Smirnov tests, as well as the randomization-based median comparison, stems from the low sample size for certain combinations of families and sets of VDOGs and the lack of normality of the distance distributions, as measured by Shapiro-Wilk tests with a significance level of 1% (see below). All synteny analyses were carried out using custom scripts written in Python 3, available upon request.

## Results

### Genomes and representative proteomes

A total of 671 herpesvirus sequences were downloaded from the NCBI genome database. After filtering entries of insufficient length, and dereplicating at the nucleotide level and protein level, 541 unique proteome clusters were obtained, 384 from the initial 2015 search and 157 from the complementary search. A summary of the distribution of all the proteome clusters among the different subfamilies, families and orders is shown in Table 1. Nineteen of these groups belong to the Alloherpesviridae, 3 to the Malacoherpesviridae and 517 to the Herpesviridae. Among the latter, 196 belong to the Alphaherpesvirinae, 265 to the Betaherpesvirinae, and 56 to the Gammaherpesvirinae. The remaining 2 proteome clusters contained viruses with unassigned family.

**Table 1:** Number of representative proteomes obtained per order, family and subfamily. The subfamily column is left blank for viral families with no subfamily as well as those with unassigned family.

Order	n	Family	n	Subfamily	n
Herpesvirales	541	Herpesviridae	517	Alphaherpesvirinae	196
				Betaherpesvirinae	265
				Gammaherpesvirinae	56
		Alloherpesviridae	19		
		Malacoherpesviridae	3		
		Unassigned (NA)	2		
Caudovirales	2045	Myoviridae	580	Eucampyvirinae	7
				Peduovirinae	30
				Spounavirinae	38
				Tevenvirinae	175
				Unassigned (NA)	330
		Podoviridae	366	Autographivirinae	101
				Picovirinae	21
				Unassigned (NA)	243
		Siphoviridae	1075		
		Unassigned (NA)	24		

For the Caudovirales, 2045 proteome clusters were generated: 580 Myoviridae, 1075 Siphoviridae and 366 Podoviridae, the rest being unclassified members of the order. Considering the subfamilies of Myoviridae, 7 groups of proteomes were composed of members of the subfamily Eucampyvirinae, 30 of the Peduovirinae, 38 of the Spounavirinae, and 175 of the Tevenvirinae. For the subfamilies of Podoviridae, 101 proteome clusters were associated to the Autographivirinae and 21 to the Picovirinae. Under the 2015 ICTV taxonomy release, Siphoviridae had no subfamilies^29^ (Table 1).

### Prevalent VDOGs

Only 5 VDOGs fulfilled the criteria established for the definition of prevalent VDOGs (see methods), and were contained in 5 viral peptides associated to 4 proteins: (i) the ATPase subunit of the DNA terminase (VDOG 105); subunits (ii) alpha (VDOG 53) and (iii) beta (VDOG 821) of the ribonucleotide reductase; (iv) the catalytic subunit of the DNA polymerase (VDOG 72); and (v) the dUTP-diphosphatase (VDOG 731). Table 2 summarizes the proteins identified, associated clusters, function, and their group as classified by McGeogh *et al*. when defining the Herpesviridae core genome^11^. With the exception of VDOG 105, which is present exclusively in the Herpesvirales and Caudovirales, the other clusters mentioned are also found in certain dsDNA viral families (Supplementary Table 1), particularly in the Phycodnaviridae. However, no other viral genome contained all 5 prevalent VDOGs apart from Herpesvirales and Caudovirales. Moreover, all five prevalent VDOGs were also deemed representative (see below). Hence, these five peptides, belonging to four proteins, are proposed to compose the core genome of the Herpesvirales.

**Table 2:** Description of the four proteins related to the prevalent Herpesvirales viral domain orthologous groups (VDOGs).

Protein	VDOG(s)	Function	Group in McGeoch *et al*.^11^
DNA terminase, ATPase subunit	105	Cleavage of concatemeric DNA during DNA packaging^14^.	Processing and packaging of DNA.
Ribonucleotide reductase	53 (alpha subunit); 821 (beta subunit)	Catalysis of the synthesis of deoxyribonucleotides from ribonucleotides^39,46^.	Enzymes peripheral to DNA replication.
DNA polymerase	72	DNA synthesis. Can cleave DNA-RNA and DNA-DNA heteroduplexes^47^.	DNA replication machinery.
dUTP-diphosphatase	731	Control of the ratio of dUTP to dTTP, to prevent the first from being incorporated to the DNA^48^.	Enzymes peripheral to DNA replication.

Also shown is the protein name, VDOG id, function of the protein, and its classification under the categories defined by McGeogh *et al*.^11^.

**Figure 1:**
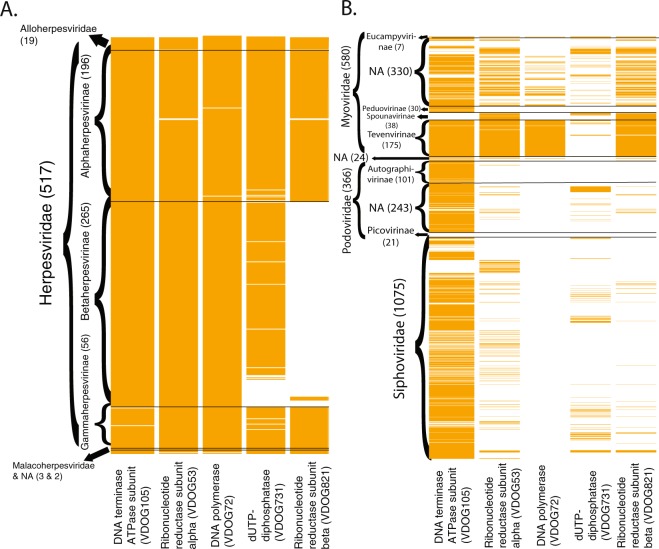
Heatmap for the presence/absence of the 5 prevalent VDOGs in the Herpesvirales (**A**) and Caudovirales (**B**). Presence (orange) or absence (white) of the 5 VDOGs (columns) from 4 different viral proteins are shown for individual proteomes (rows; n = 541 for the Herpesvirales and 2045 for the Caudovirales) sorted by NCBI taxonomic classification. Families and subfamilies are shown on the left along with the total number of proteomes depicted in each category in parenthesis.

A heatmap of presence/absence of these groups, both in the Herpesvirales (Fig. 1A) and Caudovirales (Fig. 1B) was generated. Representative proteomes are sorted based on the NCBI taxonomy of their associated genome entry. As expected, the most prevalent orthologous protein cluster is related to the ATPase subunit of the DNA terminase: in Herpesvirales, it is harboured by all but 4 of the 541 representative proteomes, the exceptions being 2 members of the Herpesviridae subfamily Gammaherpesvirinae and 2 of the family Alloherpesviridae. For the Caudovirales, the orthologous protein cluster was present to some degree in all families and subfamilies, with the exception of the Podoviridae subfamily Picovirinae and the Myoviridae subfamily Spounavirinae. No other VDOGs were prevalent in the Caudovirales as a whole, although VDOGs 53, 72 and 821 are ubiquitous in the Tevenvirinae subfamily of the Myoviridae.

Notably, VDOG 821 is absent in the subfamily Betaherpesvirinae. This can be explained considering that some of the core proteins proposed for the family Herpesviridae are absent in the subfamily Betaherpesvirinae^11^ including the beta subunit of the ribonucleotide reductase. For this reason, the threshold for presence of prevalent VDOGs was lowered to 40% of proteomes in the Herpesviridae in that particular case, as compared to the standard 60%.

### Synteny analysis

**Figure 2:**
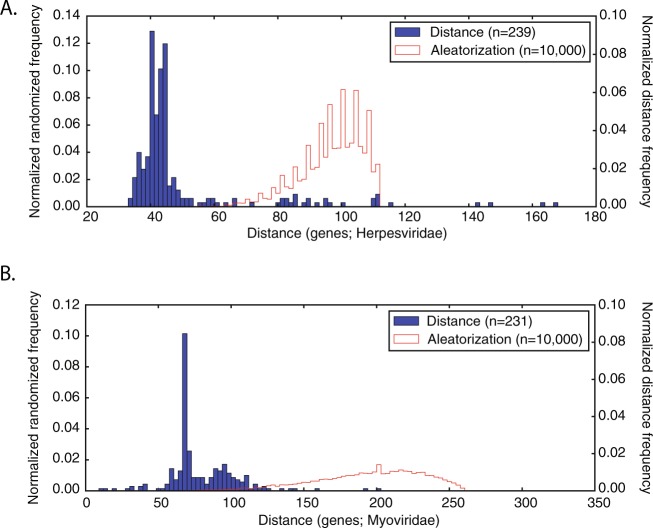
Synteny among the 5 prevalent VDOGs. Comparison of the normalized distribution of the distance, in genes, between the genes associated to the four proteins of the proposed core genome (VDOGs 53, 72, 105, 731, and 821), and a null model created from a randomization for the Herpesviridae (**A**). For the Myoviridae (**B**), the genes of the ribonucleotide reductase (alpha and beta subunits), DNA terminase, and DNA polymerase (VDOGs 53, 72, 105, and 821) are compared to the null distribution. In the inset box, *n* corresponds to the number of representative proteomes analyzed (for the real distribution; blue line); and the total number of iterations n = 10,000 for the null distribution (red line).

The genes associated to the five peptides (four proteins) proposed for the core genome were grouped at a distance significantly smaller than the one predicted by the null distribution from the 239 Herpesviridae genomes that contained them: p-value < 1e-18 for both Mann-Whitney U and Kolmogorov-Smirnov tests, median of the distance (43) smaller than the 1^st^ percentile (68) of the null distribution (Fig. 2A). Among the Caudovirales, only 16 genomes from the Myoviridae contained all Herpesvirales signature VDOGs (Supplementary Table 1). Both Mann-Whitney U and Kolmogorov-Smirnov tests yielded significant results (p-values of 2e-10 and 3e-12 respectively), and the median of the observed distances (78) was smaller than the 1^st^ percentile of the distribution generated through randomization (136).

On the other hand, for the Alloherpesviridae and Malacoherpesviridae (the other two Herpesvirales families) 16 of 19 and 3 of 3 of the genomes contained all the VDOGs respectively (Supplementary Table 1). For the Alloherpesviridae, the Mann-Whitney and Kolmogorov-Smirnov tests rejected the null hypothesis (p-values of 6e-10 and 1e-9 respectively), but that was not the case for the randomization (observed median of distance was 74, while 1^st^ percentile of null distribution was 63). Conversely, for the Malacoherpesviridae, only the Kolmogorov-Smirnov test generated significant results (p-value of 0.0079), most likely due to the small number of available genomes (n = 3). This shortage of reference proteomes, along with the fact that no set of VDOGs had a distribution which was consistently normal in all families studied under a Shapiro-Wilk test, with a significance level of 1% (Supplementary Table 2), were the reasons for the choice of the non-parametric tests previously described.

In the Myoviridae and Herpesviridae, at least two out of three tests performed were significant for all combinations of three and four VDOGs. This was also the case for the majority of combinations in the Alloherpesviridae, with 4 exceptions: VDOGs 53, 72, and 105, VDOGs 53, 72, and 821, VDOGs 53, 105, and 821, VDOGs 53, 72, and 105, and VDOGs 72, 731, and 821 (Supplementary Table 2). Interestingly, even though only 16 Myoviridae genomes harboured all five VDOGs, 231 contained VDOGs 53, 72, 105, and 821, and show evidence of synteny based on all three analyses carried out (Fig. 2B).

### Dendrogram of representative VDOGs

The procedure followed for detecting representative VDOGs for the two orders analysed, as well as their constituent families, yielded a total of 779 orthologous protein clusters. More specifically, 319 VDOGs were representative both at the order and family level, 20 exclusively at the order level, and 440 exclusively at the family level. All 5 prevalent Herpesvirales VDOGs described above were also deemed representative.

**Figure 3:**
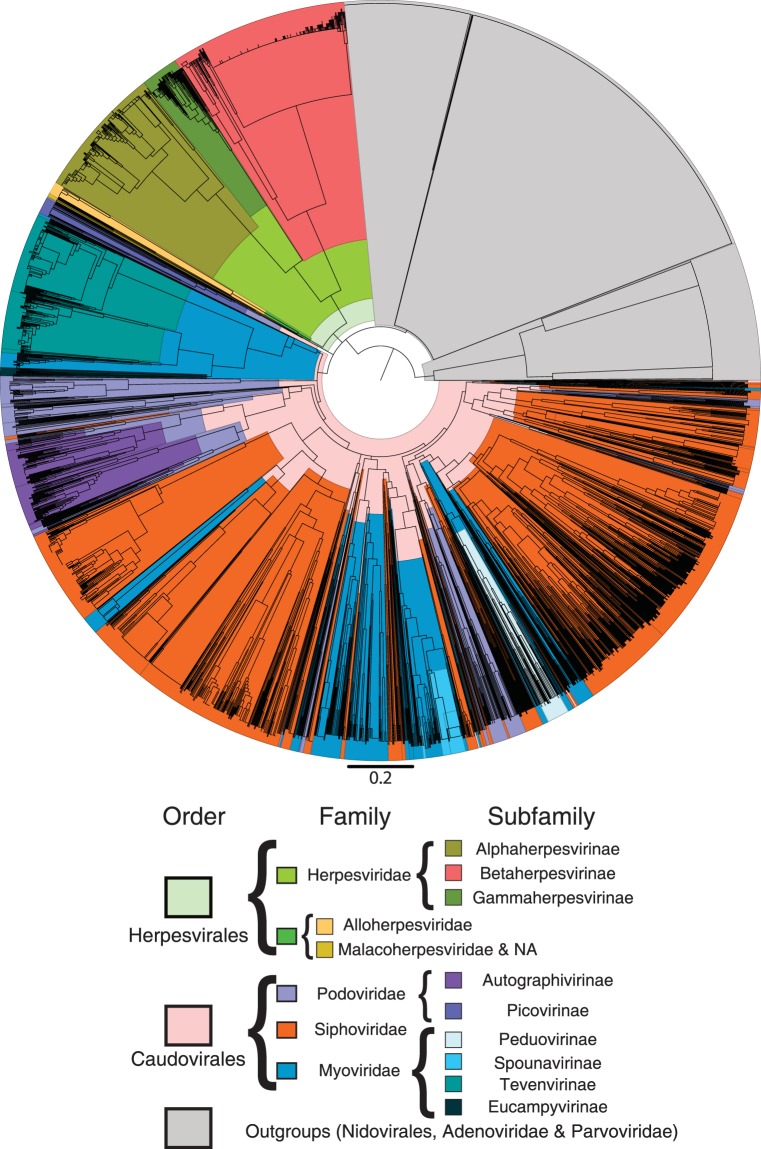
Dendrogram of presence/absence of the representative VDOGs (n = 779) in the selected proteomes of the Herpesvirales (n = 541) and Caudovirales (n = 2039). Clades are colored according to the key shown below the graph, where colors located closer to the root indicate higher taxonomy levels. Species without an assigned subfamily have only their family and order colors shown. For the Caudovirales families and subfamilies, a region colored in a specific tone implies that the majority, but not necessarily all of the representative proteomes in that branch belong to the correspondent family or subfamily. Here, a majority implies more than 70% of the proteomes in a given region. On the contrary, for the Caudovirales order, and for the Herpesvirales order and all its families and subfamilies, all regions with a given color contain exclusively proteomes from the associated clade.

For the dendrogram (Fig. 3) no outgroups with prokaryotic hosts were used since any widespread process of HGT with the Caudovirales would obscure the real evolutionary relationship, if any, between that order and the Herpesvirales. That being said, it is expected that an outgroup concordant with the hypothesis of an evolutionary relationship between Herpesvirales and Caudovirales should fulfil two conditions: be an outside branch of both of the analysed clades and have a poor internal resolution between its proteomes. Conjointly, this would mean not only that the two orders of interest are more evolutionary related between themselves than with the outgroup, but also that the representative VDOGs for the two clades analysed are not representative for the outgroup. This is precisely what is observed: members of the three outgroups, which are located in two external branches, show a poor resolution, as evidenced by the lack of internal branching. A total of 9 Caudovirales proteomes were located within these branches of the dendrogram (Supplementary Table 3): among them, 3 were entries from the *nucleotide* database, none with a supporting publication and 2 unverified, while other 3 were unverified *RefSeq* entries, so they were all removed. The remaining 3, however, were 2 unclassified Myoviridae and 1 unclassified Siphoviridae with entries supported by publications^37,38^, and thus were retained. Those viruses were located in the Nidovirales branch and the Adenoviridae branch, respectively.

Apart from those, all the Herpesvirales and Caudovirales proteomes were clustered in branches composed of members of their same order. For the Herpesvirales, which are clustered in a single branch, the classification at the family level is also consistent, with the three families forming monophyletic groups. Interestingly, the branch associated with the Malacoherpesviridae also harbours the two proteomes without family classification. This group, along with the Alloherpesviridae, forms a sister clade to the Herpesviridae. Inside the latter, the Gamma- and Alphaherpesvirinae are sister clades, apart from the Betaherpesvirinae.

On the contrary, the Caudovirales form two branches, one small branch with members of the Podoviridae subfamily, Picovirinae, and a main branch with the rest of the proteomes. Both of these branches are located in the same internal branch as the Herpesvirales, apart from the outgroups. Inside the main Caudovirales branch, the branching pattern is inconsistent with the established families, since branches from all the described families are scattered along the main branch of the order. Interestingly, the classification of subfamilies is reasonably consistent, as shown in Fig. 3, with most of the proteomes associated to each of the Caudovirales subfamilies, including the Picovirinae, either located within the same branch of the dendrogram or in neighbouring branches along with other proteomes of unassigned subfamily.

### Phylogenetic tree of the Herpesvirales

Just as the core genome proposed for the Herpesviridae included sequences conserved among its subfamilies^11^, a core genome for the whole order must include proteins with some degree of conservation among the families. In other words, peptides shared by the common ancestor of the Herpesvirales. With a sufficiently, but not excessively, high mutation rate associated to these peptides, a phylogenetic tree constructed based on their sequences should show at least the basic evolutionary relationships within the group.

**Figure 4:**
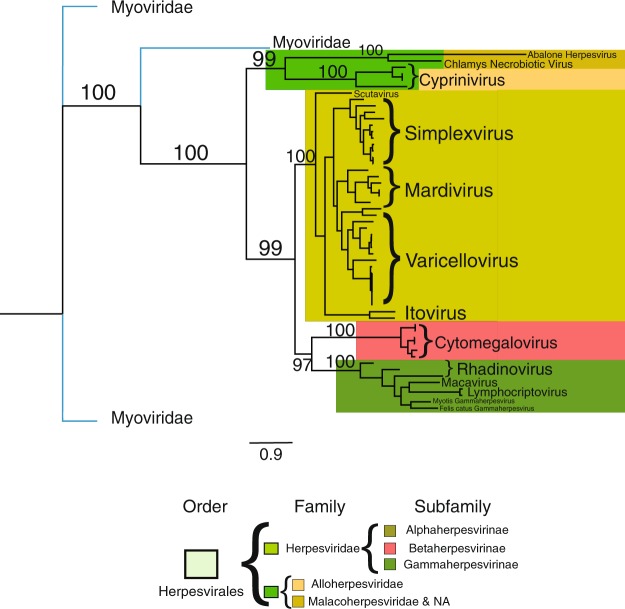
Maximum-likelihood phylogenetic tree of the Herpesvirales, constructed with the sequences of VDOGs 53, 72, 105 and 731. Outgroups are not highlighted, but their branches are colored blue. Numbers above the main branches represent bootstrap values. Clades are colored according to the key shown below the graph. Names on the trees highlight viral genera (Cyprinivirus, n = 4; Itovirus; n = 2; Mardivirus, n = 6; Scutavirus n = 1; Simplexvirus, n = 11; Varicellovirus, n = 16; Cytomegalovirus, n = 6; Lymphocriptovirus, n = 2; Macavirus, n = 1; Rhadinovirus, n = 3) or species employed (Felis catus gammaherpesvirus 1, n = 1; Myotis gammaherpesvirus 8, n = 1; Chlamys acute necrobiotic virus, n = 1; Abalone herpesvirus Victoria, n = 1). The specific proteomes used are indicated in Supplementary Table 3.

The phylogenetic tree was constructed with four sequences per proteome, those associated with VDOGs 53, 721, 105 and 731 (Fig. 4). VDOG 821 had to be removed due to its absence in almost all the Betaherpesvirinae. The positions of the 3 Myoviridae species are consistent, all of them being in the outermost branches. As in the dendrogram, the families and subfamilies are well defined and constitute monophyletic groups. Again, the unclassified and Malacoherpesviridae proteomes used here are grouped together, being a sister clade of the Alloherpesviridae. The only notable difference with the dendrogram in Fig. 3 is in the arrangement of the Herpesviridae subfamilies: The Beta and Gammaherpesvirinae are sister clades, apart from the Alphaherpesvirinae. All the main branches are supported with bootstrap values higher than 95, and the overall classification produced, except for a few cases, is also consistent at the species level.

## Discussion

Among the different proteins that make up the proteomes of the Herpesvirales, those with higher variation, and thus less conservation, usually mediate the interaction between the virus and its host^6^. As such, it would be expected that the core genome of the order would be composed mostly, or completely, of peptides related to fundamental processes in the life cycle of the members of this clade. This is precisely what was observed, with all peptides participating directly or indirectly in nucleotide synthesis or DNA packaging. The final set of core genes includes the ATPase subunit of the DNA terminase, the big and small subunits of the ribonucleotide reductase, the catalytic subunit of the DNA polymerase, and the dUTP-diphosphatase. The beta subunit of the ribonucleotide reductase is considered a part of the core genome of the family Herpesviridae^11^, even though it is either absent or dysfunctional in the subfamily Betaherpesvirinae^39^. For this reason, it was included as part of the proposed core genome but excluded from the phylogenetic analysis.

It is important to note that, for putative core genome sequences, we are employing the presence of a prevalent VDOG as a surrogate marker of its function. This is validated by the fact that over 99% of the annotated sequences composing each of the core genome VDOGs have conserved functional annotation as well: more specifically, there were 2 annotated sequences out of 1998 in VDOG105 whose function was not the one indicated here (that is, ATPase subunit of the terminase), 5 out of 1002 for VDOG 53, 1 out of 845 for VDOG731, 1 out of 926 for VDOG731, and 6 out of 773 for VDOG821. Therefore, we believe that presence of the VDOG is a strong indicator of the function of the protein for these 5 specific cases.

The core genome proposed here is supported by the phylogenetic tree obtained for the order, given that all the families and subfamilies, and most of the species, form monophyletic groups with good bootstrap values (except for certain inner branches, all values were above 80). Despite this, the specific relationships between the main branches are not the ones seen in the literature, where either the Alloherpesviridae or the Malacoherpesviridae are proposed as the basal clade, with the two remaining groups being sister clades^12,40^. For the Herpesviridae, however, the relationship of the subfamilies as shown in the phylogenetic tree is exactly as proposed by Davison & McGeogh, with the Beta and Gammaherpesvirinae being sister clades^6,12^. To the best of our knowledge the phylogeny produced here is the only one constructed for the whole order using sequences from more than one protein and over 50 proteomes, so it could be the case that the single genes employed until now (particularly the ATPase subunit of the DNA terminase), do not reflect, by themselves, the evolutionary history of the entire order.

Further research is needed to verify our hypothesis for the evolution of the Herpesvirales, as attested by the phylogenetic tree. If true, and recalling the specific hosts of each of the Herpesvirales families, as well as the fact that bivalves and amphibians arose before birds, mammals and reptiles, the tree suggests that the common ancestor of the order infected either amphibians, or bivalves (or another related invertebrate). From that initial stage, one of the lineages would adapt to infect reptiles, birds and mammals, constituting the Herpesviridae. The other clade, maintaining at first its initial host, would eventually split in two: one lineage, the Malacoherpesviridae, with bivalve hosts, and the other, the Alloherpesviridae, with amphibian hosts. Furthermore, a hypothesis of continuous coevolution of the viruses with their eukaryotic host starting from a basal invertebrate and following the development of vertebrate animals can be suggested as well. It is estimated that the family Herpesviridae split into its three subfamilies roughly 400 million years ago^6^. Therefore, if this hypothesis was true, the last common ancestor of the order would have risen before the separation of the vertebrates and invertebrates.

It is also important to consider that the method employed precludes the possibility of detecting conserved regulatory sequences or non-coding (nc)RNAs in the viral orders analysed. Both long ncRNAs and microRNAs have been found in members of the Herpesvirales^11^. In the Caudovirales, at least in one case, they are employed as a mechanism to bypass abortive infections^41^. As such, the proposed core genome could be expanded through further genome analyses.

For the Caudovirales, the dendrogram in Fig. 3 shows a mostly consistent classification at the order level, with the vast majority of the proteomes being grouped in a single main branch, the only exception being the subfamily Picovirinae, which is nonetheless located in the same internal branch as the Caudovirales main branch and the Herpesvirales branch, and the 3 proteomes which clustered with the outgroups. This consistency pattern is also seen for subfamilies. However, at the family level the results were different, their members being scattered in various locations along the Caudovirales branches. A possible explanation for this stems from the fact that the genomes of the tailed phages present high mosaicism^42^, where only groups of genes coding for closely-related proteins have similar evolutionary histories. This phenomenon is caused by HGT and would group proteomes not only based on their evolutionary relationship but also on the transfer events that occurred between them (which are dependent of host range). Moreover, the classification of the three traditional Caudovirales families has come into question recently^43,44^, in further agreement with the lack of monophyly observed in our current analysis.

Whatever the case, the whole order shares a common branch with the Herpesvirales, as would be expected if there is an evolutionary relationship between them. This is supported by the external position and poor internal resolution of the outgroups. Nevertheless, this does not imply by itself that the two orders share a closer evolutionary relationship between themselves than with any other order; to prove that, the same results should be obtained in a dendrogram including all viral orders and families with a potential relation to the Caudovirales and Herpesviridae. However, it is important to recall that an initial search of the VDOGs associated with the proposed core genome proteins against other viral proteomes (those apart from Caudovirales and Herpesvirales) did not yield a single classified genome that simultaneously harboured all 5 VDOGs, and none which harboured VDOG105 (Supplementary Table 1).

There is compelling evidence in favour of the existence of a common ancestor for both orders: (i) the conservation of the ATPase subunit of the DNA terminase^6,13,14^, and (ii) similarities in structure and viral cycle that have already been mentioned^16^. Coupled to that, the aforementioned k-mer approach towards viral classification produced a dendrogram placing the Herpesvirales within the Caudovirales^17^. Another alternative methodology, the use of bipartite networks of homologous genes followed by clustering into modules, also groups these two orders together through the DNA terminase, the HK97 maturation protease, and certain capsid proteins^43^. However, the details of their relationship are more complicated. The divergent structure of the Caudovirales virion, as compared to that of other phages suggests that they do not share a common origin, or at least not a recent one; while their high diversity and abundance suggests that they are the first viral lineage to come into existence^45^. Over their long evolutionary time, this clade could have produced more than one branch that could have become a taxon with eukaryote hosts. In that case, the high similarity with the Herpesvirales would be an indicator that this is the clade that made the transition into nuclear organisms most recently. If during these hypothetical branching events, the group that retained the ancestral trait of infecting prokaryotes remained monophyletic (as opposed to the ones that made the transition to eukaryotes), then the Herpesvirales and Caudovirales would be sister clades.

Another potential explanation could be that the Herpesvirales arose from a subgroup of the Caudovirales, as has been previously suggested^16^. Some of our current results seem to support that hypothesis: (i) Between the 3 families of tailed phages, the Myoviridae, and within them the Tevenvirinae, have the highest abundance of proteomes containing the Herpesvirales VDOGs associated with the core genome proteins (Supplementary Table 1). (ii) For the concatenated phylogenetic tree, only Myoviridae proteomes could be selected as outgroups, since they were the only among the Caudovirales that simultaneously harboured the 4 orthologous clusters employed. (iii) Apart from the Herpesviridae and Alloherpesviridae and excluding the case of the Malacoherpesviridae due to the scarce number of representative proteomes, the Myoviridae is the only family for which there is some degree of evidence of synteny in the five core genes of the Herpesvirales. Additionally, the bipartite network approach mentioned above grouped the Malacoherpesviridae with a set of Myoviridae (specifically T4-like; part of the Tevenvirinae) phages, although only a single Malacoherpesviridae genome was considered for that analysis^43^.

In other words, there are independent results that suggest that the Herpesvirales arose from or are a sister clade of the Myoviridae or one of its subfamilies, most probably Tevenvirinae. Unfortunately, considering that 4 of the 5 core peptides analysed are related to proteins that are all involved in nucleotide synthesis, HGT between members of that family and the prokaryotic ancestor of the Herpesvirales cannot be discarded, as well as potential convergent evolution. As such, the evidence put forth, at least by itself, does not preclude the possibility of the two groups being sister clades.

Moreover, a number of dsDNA families related to the Tectiviridae and Polintons, particularly the giant viruses family Phycodnaviridae, harbour in most or all of their proteomes 4 out of the 5 core Herpesvirales peptides (Supplementary Table 1). Previous studies have linked the ribonucleotide reductase from the Caudovirales and Herpesvirales to that of the giant viruses^43^, suggesting a potential relationship between these two viral lineages. Further studies applying the VDOG approach should aim at tackling the possibility of a common ancestry among these groups.

## Conclusions

The use of clusters of viral domain orthologous groups, or VDOGs, could overcome the issue of high mutation rates in viral genomes and proteomes in the case analysed. Through this approach, we were able to propose for the first time a core genome for the Herpesvirales, made up of 5 peptides, which are associated with 4 proteins: small and big subunits of the ribonucleotide reductase, dUTP-diphosphatase, catalytic subunit of the DNA polymerase and ATPase subunit of the DNA terminase. While the latter acts during DNA packaging, the formers catalyse reactions related to nucleotide synthesis. The use of 4 of these 5 peptides to construct a phylogenetic tree of the order produced a consistent phylogeny in terms of the monophyletic character of families and subfamilies, but with different relationships between them compared to what has been previously described, which sheds light on the evolution of the taxon and needs to be validated in future studies.

The results obtained here indicate, in accordance with previous analyses, that there is an evolutionary relationship between the Herpesvirales and Caudovirales. While its specific nature remains elusive, hypotheses about it can be put forth. It could be the case that they are sister clades, or that the former comes from a subclade of the latter, most probably the Myoviridae or one of its subfamilies. Past work suggests that the second possibility is the most likely one, but the similarities between that family and the Herpesvirales could have arisen through ancestral events of HGT, something that should be looked into in future studies.

## Supplementary information

Supplementary Table 1, Supplementary Table 2, Supplementary Table 3

## Data Availability

All the viral genomes used in the current study are available in public databases (Supplementary Table S3). Other datasets, including the VDOGs and HMMs employed, and the metadata of the outgroups, are available from the corresponding author upon request.

## References

[1] Koonin EV, Dolja VV, Krupovic M (2015). Origins and evolution of viruses of eukaryotes: The ultimate modularity. Virology.

[2] Koonin EV, Krupovic M, Yutin N (2015). Evolution of double-stranded DNA viruses of eukaryotes: From bacteriophages to transposons to giant viruses. Ann. N. Y. Acad. Sci..

[3] Hendrix Roger W. (2008). Evolution of dsDNA Tailed Phages. Origin and Evolution of Viruses.

[4] Kristensen DM (2013). Orthologous gene clusters and taxon signature genes for viruses of prokaryotes. J. Bacteriol..

[5] Adams MJ, Lefkowitz EJ, King AMQ, Carstens EB (2013). Recently agreed changes to the International Code of Virus Classification and Nomenclature. Arch. Virol..

[6] McGeoch Duncan J., Davison Andrew J., Dolan Aidan, Gatherer Derek, Sevilla-Reyes Edgar E. (2008). Molecular Evolution of the Herpesvirales. Origin and Evolution of Viruses.

[7] Moustafa A (2017). The blood DNA virome in 8,000 humans. Plos Pathog..

[8] Virgin HW, Wherry EJ, Ahmed R (2009). Redefining Chronic Viral Infection. Cell.

[9] Virgin HW (2014). The virome in mammalian physiology and disease. Cell.

[10] Mettenleiter TC, Klupp BG, Granzow H (2006). Herpesvirus assembly: a tale of two membranes. Curr. Opin. Microbiol..

[11] McGeoch DJ, Rixon FJ, Davison AJ (2006). Topics in herpesvirus genomics and evolution. Virus Res..

[12] Davison AJ (2002). Evolution of the herpesviruses. Vet. Microbiol..

[13] Baker ML, Jiang W, Rixon FJ, Chiu W (2005). Common ancestry of herpesviruses and tailed DNA bacteriophages. J. Virol..

[14] Selvarajan Sigamani S, Zhao H, Kamau YN, Baines JD, Tang L (2013). The structure of the herpes simplex virus DNA-packaging terminase pUL15 nuclease domain suggests an evolutionary lineage among eukaryotic and prokaryotic viruses. J. Virol..

[15] Pietilä MK (2013). Structure of the archaeal head-tailed virus HSTV-1 completes the HK97 fold story. Proc. Natl. Acad. Sci. USA.

[16] Rixon FJ, Schmid MF (2014). Structural similarities in DNA packaging and delivery apparatuses in Herpesvirus and dsDNA bacteriophages. Curr. Opin. Virol..

[17] Zhang Q, Jun S-R, Leuze M, Ussery D, Nookaew I (2017). Viral Phylogenomics Using an Alignment-Free Method: A Three-Step Approach to Determine Optimal Length of k-mer. Sci. Rep..

[18] Kristensen DM, Cai X, Mushegian A (2011). Evolutionarily conserved orthologous families in phages are relatively rare in their prokaryotic hosts. J. Bacteriol..

[19] Li W, Godzik A (2006). Cd-hit: A fast program for clustering and comparing large sets of protein or nucleotide sequences. Bioinformatics.

[20] Huang Y, Niu B, Gao Y, Fu L, Li W (2010). CD-HIT Suite: A web server for clustering and comparing biological sequences. Bioinformatics.

[21] Hyatt, D. *et al*. Prodigal: Prokaryotic gene recognition and translation initiation site identification. *BMC Bioinformatics* **11** (2010). 10.1186/1471-2105-11-119 PMC2848648 20211023

[22] Borodovsky M, McInich J (1993). GeneMark: Parallel Gene Recognition for Both DNA Stramds. Comput. Chem..

[23] Overbeek R (2014). The SEED and the Rapid Annotation of microbial genomes using Subsystems Technology (RAST). Nucleic Acids Res..

[24] Zdobnov EM, Apweiler R (2001). InterProScan - An integration platform for the signature-recognition methods in InterPro. Bioinformatics.

[25] Kristensen DM (2010). A low-polynomial algorithm for assembling clusters of orthologous groups from intergenomic symmetric best matches. Bioinformatics.

[26] Eddy S (1998). Profile hidden Markov models. Bioinformatics.

[27] Eberle R, Hayward GS, Roizman B (2013). The Order Herpesvirales. Arch. Virol..

[28] Adriaenssens EM (2017). Taxonomy of prokaryotic viruses: 2016 update from the ICTV bacterial and archaeal viruses subcommittee. Arch. Virol..

[29] Adams MJ (2015). Ratification vote on taxonomic proposals to the International Committee on Taxonomy of Viruses (2015). Arch. Virol..

[30] Iglewicz, B. & Hoaglin, D. *Volume 16: How to Detect and Handle Outliers*. *The ASQC Basic References in Quality Control: Statistical Techniques* (ASQC Quality Press, 1993).

[31] Pedregosa, F. *et al*. Scikit-learn: Machine Learning in Python. **12**, 2825–2830 (2012).

[32] Edgar RC (2004). MUSCLE: Multiple sequence alignment with high accuracy and high throughput. Nucleic Acids Res..

[33] Waterhouse AM, Procter JB, Martin DMA, Clamp M, Barton GJ (2009). Jalview Version 2-A multiple sequence alignment editor and analysis workbench. Bioinformatics.

[34] Darriba D, Taboada GL, Doallo R, Posada D (2011). ProtTest-HPC: Fast selection of best-fit models of protein evolution. Lect. Notes Comput. Sci. (including Subser. Lect. Notes Artif. Intell. Lect. Notes Bioinformatics).

[35] Stamatakis A (2014). RAxML version 8: A tool for phylogenetic analysis and post-analysis of large phylogenies. Bioinformatics.

[36] Ackermann H-W (1998). Tailed Bacteriophages: The Order Caudovirales. Adv. Virus Res..

[37] Essoh C (2015). Investigation of a large collection of Pseudomonas aeruginosa bacteriophages collected from a single environmental source in Abidjan, Côte d’Ivoire. PLoS One.

[38] Villion M (2009). P087, a lactococcal phage with a morphogenesis module similar to an Enterococcus faecalis prophage. Virology.

[39] Wei M, Cheng A, Wang M (2012). The small subunit of ribonucleotide reductase gene and protein of herpes viruses. Rev. Med. Microbiol..

[40] Fournier PG, Vanderplasschen A (2011). Cyprinid herpesvirus 3: An interesting virus for applied and fundamental research. Bull. Acad. Vet. Fr..

[41] Blower, T. R., Evans, T. J., Przybilski, R., Fineran, P. C. & Salmond, G. P. C. Viral Evasion of a Bacterial Suicide System by RNA-Based Molecular Mimicry Enables Infectious Altruism. *PLoS Genet*. **8** (2012). 10.1371/journal.pgen.1003023 PMC3475682 23109916

[42] Hatfull GF, Hendrix RW (2011). Bacteriophages and their genomes. Curr. Opin. Virol..

[43] Iranzo J, Krupovic M, Koonin EV (2016). The double-stranded DNA virosphere as a modular hierarchical network of gene sharing. MBio.

[44] Aiewsakun Pakorn, Adriaenssens Evelien M., Lavigne Rob, Kropinski Andrew M., Simmonds Peter (2018). Evaluation of the genomic diversity of viruses infecting bacteria, archaea and eukaryotes using a common bioinformatic platform: steps towards a unified taxonomy. Journal of General Virology.

[45] Ackermann HW (2003). Bacteriophage observations and evolution. Res. Microbiol..

[46] Prichard M, Shipman JC (1995). Ribonucleotide Reductase: An Important Enzyme in the Replication of Herpes Simplex Virus Type 1 and a Target for Antiviral Chemotherapy. Chemotherapy.

[47] Crute JJ, Lehman IR (1989). Herpes Simplex-1 DNA Polymerase: Identification of an Intrinsic 5′-3′ Exonuclease with Ribonuclease H Activity. J. Biol. Chem..

[48] Chen MS, Walker J, Prusoff WH (1979). Kinetic studies of herpes simplex virus type 1-encoded thymidine and thymidylate kinase, a multifunctional enzyme. Kinetic Studies and Thymidylate of Herpes Simplex Virus a Multifunctional Type l-encoded Enzyme * Thymidine. J. Biol. Chem..

